# Effect of Guava Seeds on the Biochemical Parameters and Composition of HDL Subclasses in Ovariectomized Rats

**DOI:** 10.3390/antiox14101240

**Published:** 2025-10-15

**Authors:** Lisette Monsibaez Ramírez-Melo, Elizabeth Carreón-Torres, Araceli Castañeda-Ovando, Eduardo Fernández-Martínez, Óscar Pérez-Méndez, Diego Estrada-Luna

**Affiliations:** 1 Área Académica de Nutrición, Instituto de Ciencias de la Salud, Universidad Autónoma del Estado de Hidalgo, Circuito Ex Hacienda La Concepción S/N, Carretera Pachuca-Actopan, San Agustín Tlaxiaca 42160, Hidalgo, Mexico; ra312924@uaeh.edu.mx; 2 Departamento de Biología Molecular, Instituto Nacional de Cardiología Ignacio Chávez, Juan Badiano 1, Ciudad de México 14080, Mexico; juana.carreon@cardiologia.org.mx (E.C.-T.); oscar.perez.m@tec.mx (Ó.P.-M.); 3 Área Académica de Química, Instituto de Ciencias Básicas e Ingeniería, Universidad Autónoma del Estado de Hidalgo, Mineral de la Reforma 42184, Hidalgo, Mexico; ovandoa@uaeh.edu.mx; 4 Área Académica de Medicina, Instituto de Ciencias de la Salud, Universidad Autónoma del Estado de Hidalgo, Mineral de la Reforma 42184, Hidalgo, Mexico; efernan@uaeh.edu.mx; 5 Tecnologico de Monterrey, Campus Ciudad de México, Ciudad de México 14380, Mexico; 6 Área Académica de Enfermería, Instituto de Ciencias de la Salud, Universidad Autónoma del Estado de Hidalgo, Circuito Ex Hacienda La Concepción S/N, Carretera Pachuca-Actopan, San Agustín Tlaxiaca 42160, Hidalgo, Mexico

**Keywords:** HDL subclasses, lipid profile, ovariectomized, menopause, guava seeds

## Abstract

Estrogen deficiency is associated with endothelial dysfunction, vascular inflammation, increased lipoprotein oxidation, accumulation of lipid-rich material, and platelet activation. The absence of estrogen causes physiological, metabolic, and biochemical changes that increase the risk of cardiometabolic disease development caused by a deregulation in metabolic processes such as lipid metabolism and plasma lipoprotein levels. High-density lipoprotein (HDL) has cardioprotective properties related to the quality and the quantity of its components that can be modified by some nutritional factors. Guava (*Psidium guajava* L.), a widely cultivated fruit in Mexico, is notable for its high polyunsaturated fatty acid and dietary fiber content in its seeds, but its effect on health is understudied. This study aimed to evaluate the effect of guava-seed supplementation on body weight, blood pressure, lipid profile, HDL composition, and paraoxonase-1 (PON1) activity in an ovariectomized rat model (OVX). Four groups with six adult female Wistar rats each were classified as a SHAM group: rats with simulated ovariectomy; OVX group: rats with ovariectomy; OVX + GS group: ovariectomized rats supplemented with 6 g of guava seeds; OVX + DGS group: ovariectomized rats supplemented with 6 g of defatted guava seeds. Biochemical parameters, size, and lipid concentration of HDL subclasses, apolipoproteins, and PON1 activity were determined. A decrease in body weight gain, systolic blood pressure, mean arterial pressure, and triglycerides in plasma was observed at the end of the experiment in the supplemented groups. The supplementation of 6 g of guava seeds for 30 days decreased biochemical parameters in ovariectomized rats; these results could be attributed to the seed composition, suggesting a protective effect against the risk of developing diseases in menopausal states.

## 1. Introduction

The substantial reduction and deficiency in circulating levels of estrogen affects almost all organ systems, including the cardiovascular, immune, and digestive systems. In this context, menopause is characterized by depletion of the oocytes stored in the ovaries and a significant decrease in estrogen levels. Menopausal women experience significant physiological, metabolic, and biochemical changes such as an increase in total cholesterol, triglycerides, and low-density lipoprotein cholesterol (LDL-c), and a decrease in high-density lipoprotein cholesterol (HDL-c), as well as a shift in the distribution of lipid composition [1]. In this sense, estrogens and other ovarian hormones possess cardioprotective properties and play a critical regulatory role in glucose metabolism, lipid homeostasis, and inflammatory pathways [2,3], including insulin sensitivity, blood pressure, and prothrombotic processes [4].

The decrease in estrogen secretion leads to a reduced capacity of HDL to efflux cholesterol, which is mainly related to HDL core and surface lipid remodeling (free cholesterol, triglycerides, phospholipids, and sphingolipids), which has been described in women during menopause, suggesting indirect effects of estrogen signaling pathways on the lipid profile [5,6]. The lipidome and proteome have also been described to be altered with estrogen deficiency; this could potentially compromise the other cardioprotective properties of HDL. Therefore, this suggests that HDL may become dysfunctional when circulating estrogen levels are reduced, such as in a state of menopause, affecting their quality more than the quantity of these lipoproteins [7,8]. On the other hand, some nutritional factors and bioactive compounds have been shown to influence the metabolism of estrogen, HDL structure and composition, and cardiovascular health [9,10], so lowering cardiovascular risk factors during and after menopause through diet intervention is possible.

Guava (*Psidium guajava* L.) is a fruit from the Myrtaceae family and is widely cultivated in Mexico, ranking third worldwide for production [11]. During the processing of this fruit, pulp is obtained as the main product and seeds and peels as by-products [12]. Due to the presence of bioactive compounds, the pulp and the by-products have the potential to be used in healthy processed foods [13]. Guava is notable for its high vitamin C content, as well as for its seeds, for their polyunsaturated fatty acid (PUFAs) and dietary fiber content [14]. Guava seeds and oil have been characterized; however, their effect on health remains understudied. Significant improvements in lipid profile were also reported: total cholesterol, LDL-c, and triglycerides decreased, while HDL-c increased in diabetic mice supplemented with guava seed extract [15]. Also, guava seed oil has been shown to have antioxidant activity [16], anti-LDL peroxidation, and Gram-negative-bacteria inhibitory activity [17,18].

Therefore, in the present study, we aimed to evaluate the effect of guava-seed supplementation on body weight, blood pressure, lipid profile, HDL composition, and PON1 activity in an ovariectomized rat model.

## 2. Materials and Methods

### 2.1. Animals

Twenty-four adult female Wistar rats, weighing 200–250 g and aged 3 months, were maintained under temperature-controlled conditions with light–dark cycles (12 h) and received commercial feed (LabDiet 5008^®^, LabDiet, Richmond, IN, USA) and water ad libitum. Four groups were formed, with *n* = 6 experimental animals in each group, classified as follows: SHAM group: negative control, rats with simulated ovariectomy; OVX group: positive control, rats with ovariectomy; OVX + GS group: rats supplemented with 6 g of guava seeds; OVX + DGS group: rats supplemented with 6 g of defatted guava seeds. The treatment groups (OVX + GS and OVX + DGS) followed the same procedure as the OVX group and received a supplement of guava seeds prepared as described above and mixed with commercial LabDiet 5008 rat food in a 70–30% ratio (14 g of commercial food + 6 g of guava seeds) in pellet form for 30 days. The experimental protocols were carried out according to the Guidelines for the Protection of Animals Used for Scientific Purposes (2010/63/EU) and National Guidelines (NOM-O62-ZOO-1999) [19] and were approved by the Animal Ethics Committee of the Universidad Autónoma del Estado de Hidalgo (CICUAL/-V-I/02/2025).

### 2.2. Ovariectomy

To remove the ovaries, a dorsal ovariectomy was performed in which the rats were anesthetized for surgery using a combination of xylazine hydrochloride (2.5 mg/kg of body weight) and Zelazol^®^ (Zoetis, Mexico City, Mexico) (10 mg/kg of body weight). To remove the ovaries, an incision was made on the left side of the vertebral column of the animal, the uterine horns were identified, fixed to the ovary at one end and to the uterus at the other. Ligatures were established on both sides of the ovary, sectioning and removing them. Once the ovaries were removed, the incision was sutured. The SHAM group underwent the same procedure, but the ovaries were not removed [20,21]. After surgery, meloxicam (0.3 mg/kg of body weight) was administered for 5 days for pain management.

### 2.3. Guava Seeds

For the supplement, ripe, undamaged guavas were obtained from a local market in the city of Pachuca, Hidalgo, which are harvested in the state of Michoacan. The fruits were washed and disinfected to remove the seeds. The guava seeds were washed with purified water to eliminate any pulp that could remain attached. Once clean, the guava seeds were air-dried at room temperature for 24 h and ground in a commercial coffee grinder; a portion was used in the group OVX + GS and stored until use in darkness at room temperature.

### 2.4. Seed Delipidation

The rest of the ground seeds were used for lipid extraction with dichloromethane at a ratio of 1:10 (*w*/*v*), and the mixture was stirred at 100 rpm/35 °C for 8 h. Following this, the mixture was centrifuged at 5000 rpm for 30 min to separate the solvent and then dried in an oven at 60 °C for 40 min to remove the solvent entirely, and stored for the group OVX + DGS.

### 2.5. Blood Pressure

Blood pressure levels were measured in the tail of rats after heating by a non-invasive method, and recorded in a CODA TM system from Kent Scientific Corporation (Torrington, CT, USA); this was performed with at least six blood pressure recordings for each animal, and the data were collected using CODA TM version 4.1 software for further processing.

### 2.6. Lipid Profile

Total cholesterol, triglycerides, and phospholipids levels in plasma, as well as HDL-c, HDL triglycerides (HDL-Tg), and HDL phospholipids (HDL-PPL) levels, were determined using commercial colorimetric enzymatic methods (Randox^®^, Crumlin, UK; Wako, Ltd., Osaka, Japan). Absorbance was read at a wavelength of 505 nm for cholesterol and triglycerides and 600 nm for phospholipids in a spectrophotometer (BECKMAN COULTER DU^®^ UV/Vis Spectrophotometer, Krefeld, Germany). For the lipid composition of HDL, we used the phase containing the HDL fraction of plasma after ultracentrifugation, following the methodology described below.

### 2.7. HDL Size and Lipid Concentration of HDL Subclasses

HDL particles were separated via sequential ultracentrifugation in a Beckman Optima TLX table (Indianapolis, IN, USA) [22,23,24,25]. Total apo-B-containing lipoproteins were obtained by density < 1.063 mg/dL, whereas total HDL was 1.063 < density < 1.21 g/mL. The HDL particles were dialyzed against 0.09 M Tris/0.08 M boric acid/3 mM EDTA (TBE) buffer, pH 8.4. Then, HDL particles were separated further by their hydrodynamic diameters in non-denaturing 3–30% gradient polyacrylamide gel electrophoresis (PAGE). Gels were stained for total cholesterol, phospholipids, and triglycerides using enzymatic mixtures previously described [26,27,28]. The electrophoresis gels were then scanned (Bio-Rad GS-670 densitometer, Hercules, CA, USA) and stained again for proteins with Coomassie Blue R-250, before being scanned once more. The relative proportions of each HDL subclass were estimated via optical densitometry analysis, using as reference globular proteins (thyroglobulin, 17 nm; ferritin, 12.2 nm; catalase, 10.4 nm; lactate dehydrogenase, 8.2 nm; albumin, 7.1 nm; high-molecular-weight calibration kit, Amersham Pharmacia Biotech, Buckinghamshire, UK) [24,28]. The relative proportion of every HDL subclass is expressed as the percentage of the total HDL area under the curve, integrated from 7.9 to 12.36 nm. For the classification of the HDL subclasses, we considered the following size intervals: HDL3c, 7.94–8.45 nm; HDL3b, 8.45–8.98 nm; HDL3a, 8.98–9.94 nm; HDL2a, 9.94–10.58 nm; and HDL2b, 10.58–13.59 nm [28]. Enzymatic staining of total cholesterol, triglycerides, and phospholipids of HDL subclasses was performed according to the methodology previously described [27].

### 2.8. Determination of Apolipoproteins

Apolipoprotein (apo) composition was determined on an SDS-denaturing 4–21% PAGE gradient. Fifteen micrograms of previously isolated HDL protein was used. The bands of each apo were stained with Coomassie Blue and destained with a solution of methanol, acetic acid, and water (30/58/12, *v*/*v*/*v*). The apos were identified by comparison with molecular mass standards and subsequently analyzed by optical densitometry. The results are expressed as the percentage of each apo relative to total HDL protein. The apos CI and CII are expressed as a percentage of apo C [29].

### 2.9. Paraoxonase-1 Activity

PON1 was determined using the method described by Gan et al. [30]. The trial mixture included 1 mM phenylacetate as substrate and 0.9 mM CaCl_2_ in 20 mM Tris HCl, pH 8, and 10 μL of rat plasma heparin (diluted 1:10). Enzymatic hydrolysis of the substrate was measured spectrophotometrically at 270 nm (UVVIS Beckman Coulter, Brea, CA, USA). The absorbance at 270 nm for the reaction was 1310 M^−1^ cm^−1^. Enzyme activity was expressed as the number of micromoles of phenylacetate hydrolyzed per minute per milliliter of plasma.

### 2.10. Data Analysis

Normal distribution of all data was verified using the Shapiro–Wilk test. Since these data were normally distributed, they are presented as mean ± standard deviation (SD). Differences between groups were determined using analysis of variance (ANOVA) and post hoc analysis (Tukey’s test). A *p*-value < 0.05 was considered statistically significant. All analyses were performed using SPSS version 24 software (SPSS Inc. IBM, Chicago, IL, USA).

## 3. Results

### 3.1. Biochemical Parameters

Table 1 shows the biochemical changes observed after supplementation, with a final body weight decrease of 50% in the OVX + GS group compared to the OVX group. The OVX + DGS group showed a significantly lower blood pressure level than the OVX group. In the same direction, plasma glucose levels decreased with guava-seed supplementation: 38.2% in the OVX + GS group and 57% in the OVX + DSG. Concerning the results of the lipid profile in plasma of the supplemented groups compared to the OVX group, the OVX + DGS group had 48.4% fewer triglycerides and 40.2% fewer phospholipids, whereas in the OVX + GS group, there were significant reductions of 32% for triglycerides and 25.7% for phospholipids. In addition, there was a significant increase of 68.9% in non-HDL-c in the OVX + DGS group vs. the OVX + GS group.

HDL particles mainly contain phospholipids, free cholesterol, esterified cholesterol, and triglycerides. In this context, our results showed that supplementation with guava seed was significantly associated with a 60% reduction in c-HDL plasma levels in the OVX + DGS group than in the other two groups. We further determined the HDL-Tg/HDL-PPL and HDL-c/HDL-PPL ratios as markers of HDL lipid composition [26]. The results of the OVX + DGS group showed a significant reduction of 20.5% in the HDL-c/HDL-PPL ratio after supplementation (Table 2).

### 3.2. Size and Lipid Composition of HDL Subclasses

HDL is a mixture of different particles, which, depending on their lipid and protein composition, may impact the biological function and structure of these lipoproteins. Thereby, after 30 days of supplementation, changes were found in the size and composition of HDL subclasses. Significant reduction was observed in the protein composition of the HDL3c subclass in the OVX + DGS group compared to the OVX + GS group (Table 3). Moreover, an increase in cholesterol was found in the OVX + DGS group in the small subclasses 3b and 3c compared to the OVX group. According to the composition of HDL triglycerides and phospholipids, no significant differences were observed between groups, except for an increase in phospholipids in the OVX + DGS group compared with the OVX + GS group.

### 3.3. Apolipoproteins

The apolipoprotein composition (protein fraction of HDL particles) in the experimental groups is listed in Table 4, showing a significant decrease in apo E content and a significant increase in apo C concentration in the OVX + DGS group compared to the other test groups.

### 3.4. PON1 Activity

**Figure 1:**
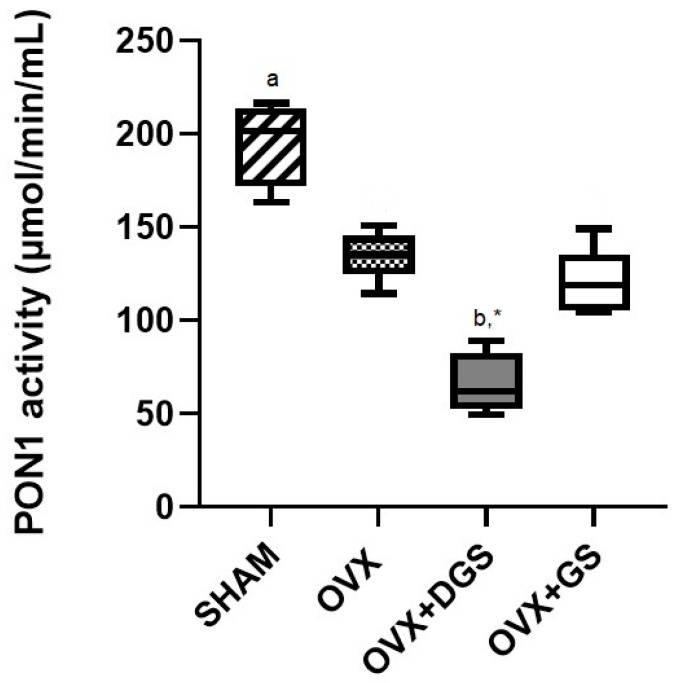
Paraoxonase-1 activity in rat plasma. Data are expressed as median (horizontal lines) and interquartile range (boxes). *n* = 6. SHAM: SHAM-operated; OVX: ovariectomized; OVX + DGS: ovariectomized + defatted guava seeds; OVX + GS: ovariectomized + guava seeds. Tukey’s test (*p* < 0.05). OVX vs. ^a^ SHAM; OVX vs. ^b^ OVX + DGS; OVX + GS vs. * OVX + DGS.

Finally, the antioxidant activity of PON1-dependent HDL is modified by the structural and chemical composition of these lipoproteins. Therefore, we determined the antioxidant activity of PON1. Interestingly, we found a significant decrease in the OVX + DGS group compared to the other test groups. Likewise, there was a significant decrease in the OVX group compared to the SHAM group (Figure 1).

## 4. Discussion

In the present study, we determined the effect of guava seed consumption in an ovariectomized animal model by simulating the biological processes due to a reduction in estrogen levels. In this model, estrogen depletion is related to metabolic effects and significant changes in body composition, including visceral fat accumulation, glucose and lipid metabolism disorders, and increased blood pressure, factors that significantly influence the high risk of developing cardiovascular diseases (CVD) [31].

An increase in body mass was observed in ovariectomized rats; nevertheless, a lower increase in body weight was observed in OVX + GS rats. A similar effect to this study was also found after administration of chia seed powder [32], as well as in the supplementation of pumpkin seed extract—high in omega-6 content [33]; in both studies, the effects are attributed to the high content of PUFAs that can help to reduce obesity by ameliorating oxidative stress, suppressing appetite, improving lipid oxidation and energy expenditure, as well as reducing fat deposition [32,33]. Therefore, in this work, PUFAs also could be responsible for the reduction in weight gain, given that the group supplemented with defatted seeds did not show significant differences compared to the ovariectomized group without supplementation.

In this sense, it has been mentioned that the composition of guava seed is highlighted by a content of fatty acids: 79% linoleic, 8% palmitic, 7% oleic, and 5% stearic [14]. Phytosterols have also been reported in guava seed oil, predominantly β-sitosterol (297.61 mg/100 g), stigmasterol (0.22 mg/100 g), and campesterol (11.04 mg/100 g) [16]. Also, they contain between 64 and 67% total dietary fiber, which comprises ~0.4% soluble dietary fiber, while insoluble dietary fiber covers most with 46–63% [14,34,35]. The last component may have an impact on improving blood pressure, but the mechanisms are not totally clear. Nevertheless, it has been suggested that these mechanisms could include a reduction in inflammation levels [36,37] and an improvement in endothelial function [38]. Additionally, studies have shown that long-term consumption of high-fiber diets improves glucose tolerance. Cellulose has been shown to inhibit starch digestion by binding to α-amylase [39], reducing glucose absorption, enhancing insulin sensitivity, and, as a result, lowering the risk of hypertension [40].

On the other hand, in the oil extraction process, a by-product obtained is protein paste. After the seeds were defatted, the guava seed paste had a higher content of protein (9.9% d.b.) and essential and nonessential amino acids (mainly leucine, arginine, glutamic acid, proline, among others), 12.0% (d.b.) of carbohydrate, a lower fat content (2.4% d.b.) and the highest values of raw fiber (69.3% d.b.) [41,42], this composition may be associated with the biological effects found in this study.

Plasma lipid depletion in rats from the OVX + DGS and OVX + GS groups suggests a reduction in hepatic cholesterol and triglyceride synthesis [43], as well as an increase in intestinal transit by insoluble fibers, which can create a physical barrier and, as a result, a decrease in lipid absorption [44].

Our results of the composition of HDL subclasses show a decrease in HDL 3c size in the OVX + DGS group in contrast to the OVX + GS group. In addition, there was an increase in the concentration of cholesterol in the smaller subclasses (HDL 3b and 3c) with respect to the OVX group, without observing significant changes in the composition of triglycerides between groups. A larger number of small HDL particles and their cholesterol efflux capacity could be related to the lowest HDL-c levels in the OVX + DGS group. Dietary intake is an important and modifiable factor that directly provides lipid precursors and modulates metabolic pathways. In particular, fatty acids and lipid-rich foods significantly alter lipid profile by changing the composition and metabolism of various species of lipids, such as triglycerides, sphingolipids, and PUFAs [45,46]. Also, diseases characterized by increased inflammatory processes are associated with changes in the lipidome of HDL, particularly a decrease in HDL phospholipid content and an increase in HDL triglycerides [47]. In a clinical study of menopausal women, HDL subfractions were evaluated; the results showed that, during the first 2 years after the decrease in estrogen secretion, the concentration and size of HDL2 subclasses decreased, while HDL3 and HDL-Tg increased [48]. There is controversy regarding whether the larger particles (HDL2) or the smaller ones (HDL3) are more atheroprotective. It has been suggested that the large HDL subclass is more prone to oxidative modification with respect to the small subclasses [49], whereas HDL3 plays a central role in the reverse cholesterol transport (RCT) by removing cholesterol from the periphery and maturing into HDL2 particles through progressive lipidation by the action of lecithin–cholesterol–acyltransferase (LCAT) [50].

Apolipoproteins are the most abundant group of proteins in HDL. In this work, the OVX + DGS group obtained the highest content of apo C and the lowest content of apo E in contrast to the other groups. Apo E is considered an atheroprotective protein because it removes more saturated than unsaturated lipids, and atherogenic plaques are rich in saturated lipids and cholesterol [51]. Likewise, apo E genotypes are viewed as key genetic determinants of inter-individual variations in postprandial lipemia. This interaction depends on the presence of certain fatty acids, such as PUFAs and phospholipids [52]. This is consistent with the significant changes observed in relation to the decrease in phospholipids and increase in non-HDL-c results in the plasma of this work. Furthermore, HDL particles containing apo E promote the efflux of cholesterol from extrahepatic cells [53] through ABCA1- and ABCG1-dependent processes, and this process is antagonized by the presence of apo CIII, which can negatively affect the antiatherogenic properties of HDL [54,55]. In a study where supplementation with PUFAs in rats was evaluated, a decrease in apo CIII was found, indicating a possible effect of these compounds on apolipoprotein gene expression [56].

It has been shown in some animal models that PON1 can prevent the harmful effects of oxidative stress on serum, and that serum levels of PON1 correlate with levels of HDL and apolipoprotein AI, although this correlation is not strong. We might suggest that increased PON1 activity is a consequence of higher HDL levels. In our study, the lower value of PON1 activity in the ovariectomized groups compared to SHAM rats can be attributed to estrogens, given that estradiol enhances PON1 activity [57]. In addition, a pro-oxidative environment could lead to an increase in the binding of free radicals to PON1, resulting in a less active enzyme in circulation [58]. It has been reported that dietary factors have a significant effect on PON1 activity, specifically polyunsaturated fatty acids [59], which could be associated with higher activity in the OVX + GS group compared to the OVX + DGS group. Furthermore, according to the observed results of apo and PON activity, the OVX + DGS group could be a population at risk for the development of cardiovascular disease.

Differences shown between the OVX + DGS and OVX + GS groups are related to the lipid composition of the seed. When supplementing with a defatted seed, the protective properties previously described were lost, indicating that such properties are associated with polyunsaturated fatty acids [60]—and probably other lipidic molecules contained in the whole seed, such as phytosterols [61]—which have been reported to benefit health owing to their cholesterol-lowering and anti-inflammatory effects [62,63]. Studies on biomodels fed a high-fat diet have proven the efficacy of supplementation with defatted seeds such as safflower (50–100 mg/kg of weight/day) [64] and poppy (33% fiber and 27% protein) [65] on plasma and hepatic lipid levels, reducing triglyceride and cholesterol levels. In addition, it has been described that defatted grape seed affects the 3T-L1 pre-adipocyte cells, showing a significant decrease in lipid accumulation, possibly due to a regulation of the mRNA expression of leptin and lipoprotein lipase (LPL), both of which have been demonstrated to regulate lipid metabolism [66].

## 5. Conclusions

The supplementation of guava seeds during 30 days limited increases in body weight, blood pressure, glucose, triglycerides, and non-HDL-c in ovariectomized rats. These results could be attributed to lipidic components and insoluble dietary fiber in the seed, suggesting a discrete protective effect against the risk of developing diseases during physiological cessation of estrogen secretion. Limitations of this study include the lack of estrogen level measurements, which future research could address, and the need for additional studies in order to further understand the role of whole and defatted guava seeds.

## Figures and Tables

**Table 1:** Biochemical parameters post-supplementation in rats.

Parameter	SHAM	OVX	OVX + GS	OVX + DGS
Glucose (mg/dL)	165.77 ± 33.54	201.59 ± 35.04 ^a,b,c^	124.50 ± 23.90	86.66 ± 11.71 *
Body weight gain (g)	28.00 ± 9.57	63.00 ± 25.16 ^a,c^	32.83 ± 3.82	57.20 ± 23.73
Systolic blood pressure (mmHg)	145.20 ± 10.60	166.94 ± 9.35 ^b^	149.38 ± 10.80	138.89 ± 17.78
Media blood pressure (mmHg)	121.30 ± 6.50	144.31 ± 10.35 ^a,b^	122.70 ± 12.01	102.42 ± 21.22
Cholesterol (mg/dL)	37.01 ± 8.42	40.88 ± 5.59	32.42 ± 6.18	33.77 ± 2.84
Triglycerides (mg/dL)	49.42 ± 14.62	55.63 ± 10.04 ^b,c^	37.83 ± 6.95	28.72 ± 5.25
Phospholipids (mg/dL)	115.46 ± 15.67	134.29 ± 19.88 ^b,c^	99.81 ± 8.06	80.37 ± 12.88
Non-HDL-c (mg/dL)	9.15 ± 6.90	7.56 ± 3.92 ^b^	4.86 ± 4.09	15.64 ± 1.17 *

Mean ± standard deviation. *n* = 6. SHAM: SHAM-operated; OVX: ovariectomized; OVX + GS: ovariectomized + guava seeds; OVX + DGS: ovariectomized + defatted guava seeds. Tukey’s test (*p* < 0.05). OVX vs. ^a^ SHAM; OVX vs. ^b^ OVX + DGS; OVX vs. ^c^ OVX + GS; OVX + GS vs. * OVX + DGS.

**Table 2:** HDL lipid profile post-supplementation in rats.

HDL Lipid Profile	SHAM	OVX	OVX + GS	OVX + DGS
HDL-c (mg/dL)	27.86 ± 4.59	33.32 ± 2.42	27.55 ± 6.90	18.13 ± 2.31 ^a,^*
HDL-Tg (mg/dL)	11.65 ± 3.18	10.42 ± 1.88	7.32 ± 2.57	8.48 ± 1.35
HDL-PPL (mg/dL)	58.80 ± 13.86	64.71 ± 4.38	61.66 ± 12.68	52.06 ± 5.31
HDL-c/HDL-PPL ratio	0.44 ± 0.08	0.52 ± 0.05	0.52 ± 0.06	0.35 ± 0.04 ^a^
HDL-Tg/HDL-PPL ratio	0.18 ± 0.09	0.16 ± 0.04	0.14 ± 0.04	0.17 ± 0.03

Mean ± standard deviation. *n* = 6. SHAM: SHAM-operated; OVX: ovariectomized; OVX + GS: ovariectomized + guava seeds; OVX + DGS: ovariectomized + defatted guava seeds. Tukey’s test (*p* < 0.05). OVX vs. ^a^ OVX + DGS; OVX + GS vs. * OVX + DGS.

**Table 3:** Size and composition of HDL subclasses.

HDL Subclasses	SHAM	OVX	OVX + GS	OVX + DGS
Protein (%)				
HDL 2b	40.60 ± 3.32	46.56 ± 2.08	43.19 ± 5.06	48.12 ± 1.64
HDL 2a	13.06 ± 2.46	12.03 ± 1.20	12.23 ± 2.28	13.26 ± 2.12
HDL 3a	16.68 ± 2.00	14.39 ± 1.06	15.10 ± 2.11	14.84 ± 1.21
HDL 3b	8.25 ± 0.92	8.23 ± 0.73	8.29 ± 1.14	7.48 ± 1.21
HDL 3c	19.00 ± 3.10	18.51 ± 1.74	21.19 ± 3.75	16.31 ± 1.07 *
Cholesterol (%)				
HDL 2b	40.53 ± 1.36	43.46 ± 2.33	39.74 ± 2.93	36.82 ± 1.60
HDL 2a	12.09 ± 0.53	11.77 ± 0.44	11.79 ± 0.82	11.04 ± 1.38
HDL 3a	17.05 ± 1.42	15.65 ± 2.10	17.19 ± 1.63	19.71 ± 4.05
HDL 3b	9.24 ± 0.80	8.42 ± 1.19	9.77 ± 1.29	11.38 ± 1.91 ^a^
HDL 3c	21.09 ± 3.20	20.69 ± 2.26	23.83 ± 2.52	27.18 ± 1.30 ^a^
Triglycerides (%)				
HDL 2b	34.90 ± 3.37	36.94 ± 2.34	36.50 ± 2.75	39.53 ± 3.08
HDL 2a	11.13 ± 1.13	11.15 ± 0.65	10.58 ± 1.28	11.75 ± 0.53
HDL 3a	17.63 ± 2.57	17.29 ± 1.58	17.05 ± 1.42	16.53 ± 0.44
HDL 3b	9.49 ± 0.41	9.28 ± 0.95	9.63 ± 1.19	9.00 ± 0.54
HDL 3c	26.84 ± 1.90	25.33 ± 2.93	26.25 ± 3.05	23.37 ± 2.62
Phospholipids (%)				
HDL 2b	41.34 ± 5.21	40.54 ± 3.64	41.42 ± 6.00	39.70 ± 0.94
HDL 2a	10.87 ± 0.76	10.39 ± 0.78	10.01 ± 0.88	11.97 ± 1.06 *
HDL 3a	16.57 ± 2.18	15.64 ± 1.03	15.47 ± 1.34	17.21 ± 0.39
HDL 3b	8.99 ± 1.01	9.27 ± 0.80	9.00 ± 0.78	8.92 ± 0.74
HDL 3c	22.24 ± 2.29	24.79 ± 1.64	24.11 ± 3.49	22.20 ± 0.93

Mean ± standard deviation. *n* = 6. SHAM: SHAM-operated; OVX: ovariectomized; OVX + GS: ovariectomized + guava seeds; OVX + DGS: ovariectomized + defatted guava seeds. Tukey’s test (*p* < 0.05). OVX vs. ^a^ OVX + DGS; OVX + GS vs. * OVX + DGS.

**Table 4:** Apolipoprotein percentage of the total HDL protein.

Apolipoproteins	SHAM	OVX	OVX + GS	OVX + DGS
Apo AIV	19.79 ± 1.53	18.22 ± 3.64	21.01 ± 3.12	22.76 ± 3.13
Apo E	19.92 ± 3.26	18.95 ± 1.85	21.68 ± 3.33	13.70 ± 3.38 ^a,^*
Apo AI	44.17 ± 3.20	45.60 ± 5.38	39.93 ± 8.10	41.79 ± 7.65
Apo AII	8.71 ± 1.12	8.99 ± 1.58	9.18 ± 1.30	8.34 ± 3.28
Apo C	5.89 ± 0.90	6.75 ± 0.88	8.20 ± 0.93	13.41 ± 5.03 ^a,^*

Mean ± standard deviation. *n* = 6. SHAM: SHAM-operated; OVX: ovariectomized; OVX + GS: ovariectomized + guava seeds; OVX + DGS: ovariectomized + defatted guava seeds. Tukey’s test (*p* < 0.05). OVX vs. ^a^ OVX + DGS; OVX + GS vs. * OVX + DGS.

## Data Availability

The original contributions presented in this study are included in the article. Further inquiries can be directed to the corresponding author.

## Abbreviations

The following abbreviations are used in this manuscript:

HDL	High-density lipoprotein
PON1	Paraoxonase-1
LDL	Low-density lipoprotein
SHAM	SHAM-operated rats
OVX	Ovariectomized rats
DGS	Defatted guava seeds
GS	Guava seeds
HDL-c	HDL cholesterol
HDL-Tg	HDL triglycerides
HDL-PPL	HDL phospholipids
Apo	Apolipoprotein
SD	Standard deviation
CVD	Cardiovascular diseases
RCT	Reverse cholesterol transport
LCAT	Lecithin–cholesterol–acyltransferase
E2	Estradiol
LPL	Lipoprotein lipase
